# Management of acute severe ulcerative colitis—an update for generalist and specialist clinicians

**DOI:** 10.1093/bmb/ldae006

**Published:** 2024-06-02

**Authors:** Anish J Kuriakose Kuzhiyanjal, Jimmy K Limdi

**Affiliations:** Digestive Diseases Unit, Aintree University Hospital, Lower Ln, Fazakerley, Liverpool L97AL, UK; Division of Gastroenterology-Section of IBD, Northern Care Alliance NHS Foundation Trust, Rochdale Old Rd, Bury, Manchester BL97TD, UK; Manchester Academic Health Sciences, University of Manchester, Oxford Rd, Manchester M139PL, UK

**Keywords:** ulcerative colitis, inflammatory bowel disease, corticosteroid, infliximab, ciclosporin

## Abstract

**Background:**

Acute severe ulcerative colitis (ASUC) is a potentially life-threatening medical emergency that occurs in up to 25% of patients with ulcerative colitis. Although intravenous corticosteroids remain the cornerstone of therapy, 30–40% of patients will not respond and need timely consideration of rescue therapy with (currently) either infliximab or ciclosporin or indeed colectomy, underscoring the importance of multidisciplinary care to ensure favourable outcomes for patients. We discuss the current evidence and present an approach to the management of ASUC for general and specialist clinicians caring for patients with ASUC.

**Sources of data:**

The information in this review is derived from data published in peer- reviewed academic journals and registered clinical trials.

**Areas of agreement:**

Management of acute severe colitis requires a multidisciplinary approach with early initiation with steroids and timely escalation of treatment to either medical rescue therapy or surgery.

**Areas of controversy:**

Balancing the risks of delayed surgery vs. optimizing medical therapy, including accelerated dosing schedules for biologics, remains ambiguous.

**Growing points:**

The position on newer molecules like Janus Kinase inhibitors, such as tofacitinib, is a growing area with early real-world data showing promise for steroid refractory ASUC.

**Areas timely for developing research:**

Developing predictive biomarkers and clinical risk scores for personalized rescue therapy selection is an evolving area of research.

## Introduction

Ulcerative colitis (UC) is a chronic, relapsing form of inflammatory bowel disease (IBD), characterized by mucosal inflammation in the colon and rectum with 20–35% of individuals experiencing extraintestinal manifestations like peripheral arthritis and primary sclerosing cholangitis. The aetiology remains elusive but is likely due to a combination of environmental triggers, immune dysregulation and a genetic predisposition. The incidence and prevalence continue to rise globally, currently estimated at 5 million cases worldwide.^1^

Acute severe ulcerative colitis (ASUC) is characterized by symptoms of rectal bleeding, increased stool frequency, urgency and tenesmus.^1^ Up to 25% of patients with UC require hospitalization for ASUC at some stage in their disease history. It is associated with considerable morbidity: 30–40% risk of colectomy after one or more exacerbations and 10–20% likely to need a colectomy during their first admission with ASUC, highlighting the need for surgical team involvement at admission.^2^^,^^3^

Timely identification and management of ASUC have played a crucial role in reducing mortality rates over the years, underpinning the need for a wide range of clinicians involved in emergency care, gastroenterologists and surgeons to be conversant with the principles and important considerations in the management of ASUC.^4^

## Management of ASUC

### Definition

ASUC is conventionally defined using the modified Truelove and Witts criteria (Table 1), by the presence of ≥6 bloody stools a day with symptoms of at least one marker of systemic toxicity: temperature ≥ 37.8°C, haemoglobin < 10.5 g/dL, erythrocyte sedimentation rate > 30 mm/hr, C-reactive protein (CRP) > 30 mg/dL and/or a pulse rate of ≥90 bpm.^5–7^

**Table 1:** Truelove and Witts’s criteria for hospitalization: CRP, C reactive protein; ESR, erythrocyte sedimentation rate

Parameter	Mild	Moderate	Severe
Bloody stools/day	<4	4 or more; if	≥6 and
Pulse (bpm)	<90	≤90	>90 or
Temperature (°C)	<37.5	≤37.8	>37.8 or
Haemoglobin (g/L)	>115	≥105	<105 or
ESR (mm/hr)	<20	≤30	>30 or
CRP (mg/L)	Normal	≤30	>30

### General management and investigations

**Fig. 1:**
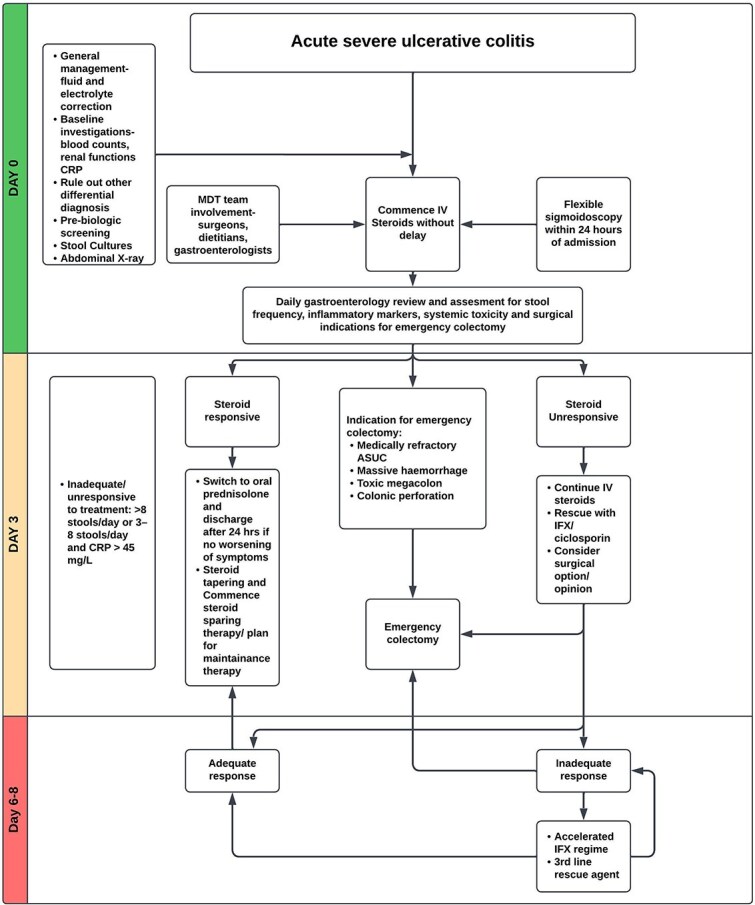
Suggested ASUC management algorithm.

Patients presenting to the hospital with ASUC require admission for intensive therapy and close monitoring to promptly identify potential complications. A comprehensive evaluation, including a detailed history of onset and a travel and drug history supported by investigations, is crucial for identifying a probable cause and excluding alternative causes of colitides (Table 2). Baseline blood tests (full blood count, CRP, electrolytes including magnesium, renal and liver function tests) are essential for optimizing initial care, including fluid resuscitation and correction of electrolyte abnormalities. Patients with ASUC tend to have hypokalaemia and hypovolaemia due to colonic losses. Moreover, hypokalaemia may precipitate colonic dilatation.^6^^,^^7^ Early stool testing with multiple samples helps differentiate UC from infectious mimics and other conditions listed in Table 2. It is imperative to note that *Clostridium difficile* exclusion would require use of a two-step testing algorithm.^8^ Pre-biologic screening should be performed as part of the initial tests to screen for viral infections and tuberculosis in anticipation of the need for rescue therapies such as infliximab (IFX) or ciclosporin. ASUC management overview is summarized in Figure 1.

**Table 2:** Differential diagnosis of ASUC

Inflammatory	Crohn's colitis
	Diverticulitis
	Segmental colitis associated with diverticulosis
	Graft vs host disease
	Radiation proctopathy
Infection	Bacterial: Aeromonas, Campylobacter, *Clostridium difficile*, *Escherichia coli*, Salmonella, Shigella, Tuberculosis, Yersinia
	Parasitic: amoebiasis, schistosomiasis
	Viral: cytomegalovirus, herpes
Drug induced	NSAID's, immune checkpoint inhibitor-induced enterocolitis, Nicorandil, Gold, antibiotics
Vascular	Ischaemic colitis, vasculitis
Miscellaneous	Postoperative diversion colitis, colorectal cancer

ASUC patients have a high risk of malnourishment due to various factors such as abdominal pain, diarrhoea and poor appetite. Nutritional assessment and optimisation is imperative for successful outcomes although there is no evidence to suggest that either bowel rest or parenteral nutrition is more effective than enteral nutrition.^9^^,^^10^ Although the use of exclusive enteral nutrition (elemental, polymeric and semi-elemental) has demonstrated a low corticosteroid failure rate and improved long-term outcomes, this is not considered as standard of care in ASUC management at present.^11^ Early dietetic team involvement is crucial to combat the risk of macro and micro nutritional deficiencies, especially due to higher perioperative adverse events in malnourished patients, should they need emergency colectomy.^12^ Furthermore, recent evidence supports an augmented response to corticosteroids with enteral nutrition and its effect on microbiome enrichment in ASUC.^9^

Antidiarrheal, anticholinergic and opioid medications should be avoided as they may increase the risk of colonic dilatation.^6^^,^^7^^,^^13^ It may be appropriate to avoid oral iron due to its postulated role on aggravating mucosal inflammation through oxidative stress from oxygen free radicals(the Fenton reaction).^14^

Before reaching a definitive diagnosis, it is imperative to consider and exclude a wide range of differential diagnoses. Stool testing helps differentiate UC from infectious mimics and other conditions listed in Table 2.

### Thromboprophylaxis

IBD is associated with an increased risk of thrombosis. While the risk of arterial thrombosis is small but noteworthy, the risk of venous thromboembolism (VTE) roughly doubles (relative risk (RR) 1.96–2.57) across various studies.^15^ VTE in hospitalized IBD patients is associated with prolonged hospital stays, increased costs and elevated mortality rates.^15^

International guidelines support pharmacological thromboprophylaxis due to its efficacy in preventing pulmonary embolism and symptomatic VTE.^6^^,^^7^^,^^13^ Low molecular weight heparin and fondaparinux are favoured over unfractionated heparin, with the former associated with lower complication rates. Direct oral anticoagulants are not recommended for primary prophylaxis during hospitalization.

While an extended duration of thromboprophylaxis after hospital discharge is not routinely advised for IBD patients, certain high-risk individuals may benefit. Factors such as older age, comorbidities, history of VTE and specific surgical procedures contribute to prolonged VTE risk after discharge. The decision for extended thromboprophylaxis should be individualized based on the patient's risk factors, as identified by validated risk models or clinical judgment, pending further validation of risk assessment models.^15^

### Antibiotics

Antibiotics are not routinely recommended in ASUC based on evidence from randomised controlled trials (RCTs) investigating adjunctive antibiotics and showing no benefit over corticosteroids.^16^ A Cochrane review by Gordan et al. found no significant difference between antibiotics and placebo when added to standard therapies for achieving short-term remission or symptom improvement.^17^ However, appropriate antibiotics may be used in case of confirmed infection, such as vancomycin for *C. difficile*.

### Imaging

Abdominal imaging, such as CT scans and plain X-rays, may be useful for assessing the extent of inflammation and ruling out complications like megacolon (colonic distention > 6 cm) and perforations in patients with ASUC. The choice of imaging modality depends on the acuity and clinical evaluation. X-ray findings may include mucosal thickening, loss of haustrations, thumb-printing and the absence of stool in the inflamed colonic segment. Visible mucosal islands, a colonic diameter > 5.5 cm, and small bowel distention > 3 cm elevate the risk of steroid failure and colectomy.^18^ If there is no clinical improvement or worsening during admission, additional imaging is recommended, especially as patients on corticosteroids may not exhibit typical features of perforation. Abdominal CT scans are typically reserved for cases with suspected extraluminal complications. In recent years, intestinal ultrasound (IUS) has emerged as a valuable non-invasive imaging modality in IBD assessments and IUS may soon assert its relevance in ASUC management. In one study, > 20% reduction in bowel wall thickness within the first 48 hr of admission with ASUC, predicted increased response to IV corticosteroids (odds ratio: 22.6; 95% CI, 4.2–201.2).^19^

### Endoscopy

Endoscopic assessment, with flexible sigmoidoscopy, is recommended within 24 hr of admission for ASUC.^6^ Flexible sigmoidoscopy by an experienced endoscopist, with minimal insufflation and without bowel preparation, while minimizing the risk of perforation, allows for confirming the diagnosis, grading the extent of inflammation and obtaining biopsies to rule out CMV colitis, especially in immunocompromised patients. A full colonoscopy is unnecessary and increases the risk of perforation.^6^^,^^7^

No standardized endoscopic scoring system specifically exists for ASUC, and although the Mayo endoscopic score is more commonly utilized, the Ulcerative Colitis Endoscopic Index of Severity (UCEIS) has demonstrated prognostic utility in this population.^20^ The presence of extensive colonic ulcerations correlates with corticosteroid non-response and superior positive predictive value (PPV) for colectomy. Specifically, a UCEIS ≥7 conveys greater colectomy predictive value compared with a Mayo score of 3.^13^

### Corticosteroid use

Corticosteroids remain the cornerstone of first-line treatment of ASUC, since the pioneering study by Truelove and Witts demonstrated substantially higher remission rates at 6 weeks with cortisone therapy (41.3%) vs. placebo (15.8%).^5^ Contemporary guidelines endorse intravenous corticosteroids equivalent to 0.8–1 mg/kg of methylprednisolone over 24 hr or 100 mg hydrocortisone four times daily for 5–7 days as initial ASUC management, with no benefit from higher dose or continuous infusion.^6^^,^^7^

Commencing or continuing aminosalicylates offers no extra benefit to hospitalized ASUC patients on IV steroids and as such, aminosalicylates may be withheld during admission, and considered if necessary at discharge.^21^ Corticosteroid administration should promptly follow clinical and endoscopic findings consistent with acute severe colitis, regardless of culture or stool assay results, to prevent complications.^6^^,^^7^^,^^13^

Though intravenous corticosteroids yield a favourable response in up to 67% of patients, colectomy rates reach up to 29% during the same admission, with a persistent 1% mortality risk, underscoring the need to identify those patients who would not respond to steroids early.^22^

Following the commencement of IV steroids, the patient should be reviewed daily, assessing stool frequency, rectal bleeding, and associated constitutional symptoms of abdominal pain, and other systemic features. Interval abdominal X-rays may be performed as per clinical need.

Various factors predict the response to corticosteroids, and these can be aided using clinical, biochemical, radiological and endoscopic parameters in addition to composite scores (Table 3).^23^ The widely employed Oxford criteria evaluate the response to intravenous corticosteroids on Day 3, considering stool frequency and CRP.^24^ Interestingly, most indices, except for the newer ‘ASUC score’ and ‘ADMIT-ASC’, necessitate a minimum of 3 days to evaluate the response, potentially impeding the early consideration of rescue therapy and surgery.^23^

**Table 3:** Scoring systems to predict response

Author (year)	Scoring system	Assessment timing	Parameters	Risk of outcome/colectomy
Deloshaan et al. (2022)	ASUC score	Day 1	1 point each for: Albumin≤30 g/L, steroid use at admission and UCEIS≥7	Score ≥ 2: PPV 92.3% for IV steroid failure
Adams et al. (2022)	ADMIT-ASC	Day 1	1 point each for: Albumin ≤ 25 g/L, CRP ≥100 mg/L and UCEIS ≥4 (or 2 points for UCEIS≥7)	Score ≥ 3: PPV 89% for IVS failure; score (IVS response rates)- 0 (100%), 1 (75%), 2 (54.9%), 3 (18.2%), 4 (0%)
Travis et al. (1996)	Oxford score	Day 3	Day 3: > 8 stools/day or 3–8 stools/day and CRP > 45 mg/L	85% colectomy risk
Berinstein et al. (2022)	IVS failure model (prior to rescue therapy))	Day 3	1 point each for: baseline IFX clearance>0.53 L/day, admission CRP > 91 mg/L, and a decrease in CRP < 43% from Day 0 to Day 3	Score (colectomy proportions in 3 months): 0 (10%), 1 (14%), 2–3 (47%)
Ho et al. (2004)	Ho index (Edinburgh score)	Day 3	Stool frequency/day (points): ≤ 4 (0); > 4 and ≤ 6 (1); > 6 and ≤ 9 (2); > 9 (4); Colonic dilatation (points): Yes (4), No (0); Albumin ≤ 30 g/L on Day 1 (1 point)	Score (same admission colectomy risk): 0–1 (11%), 2–3 (43%), ≥4 = 85%
Jain et al. (2017)	AIIMS index	Day 3	1 point each for: admission UCEIS > 6 and Day 3 FCP > 1000 μg/g	Score (PPV for steroid failure)- 0 (17%), 1 (65%), 2 (100%)
Sayal et al. (2021)	Salvage therapy response prior to salvage therapy	Day of IFX therapy	Admission albumin ≤ 2.5 g/dL and band neutrophil count ≥13% at IFX therapy	90 days colectomy risk: PPV = 100%, AUROC =0.86
Le Baut et al. (2021)	Salvage therapy response prior to salvage therapy	Discharge	1 point each for: previous anti-TNF or thiopurines, Clostridium infection, CRP > 30 mg/L, Albumin < 30 g/L on admission	Score (12-month cumulative colectomy risk): 0 (0%); 1 (9.4%); 2 (10.6%); 3 (51.2%); 4 (100%)
Jain et al. (2018)	Salvage therapy response prior to salvage therapy	Day 7	Model using IVS response on Day 7, steroid use in the first year of diagnosis, disease duration prior to ASUC presentation, and count of EIM.	Prediction accuracy of 77% for colectomy within a median duration of 56 months.

ASUC, acute severe ulcerative colitis; AUROC, area under ROC; CRP, C-reactive protein; EIM, extraintestinal manifestations; FCP, faecal calprotectin; IFX, infliximab; IVS, intravenous steroids; PPV, positive predictive value; UCEIS, ulcerative colitis endoscopic index of severity. (adapted from Kayal et al.^21^)

### Steroid-responsive patients

For patients who positively respond to initial intravenous corticosteroids ASUC, the subsequent course involves transitioning from intravenous steroids (IVS) to oral steroids and eventually tapering off steroids entirely. An optimal steroid tapering plan entails a gradual reduction in dosage over time to prevent symptom recurrence. According to a survey of 128 professionals, most healthcare providers typically prescribe a 7-day course of 40 mg oral prednisolone with a weekly reduction of 5 mg, covering a total duration of 8 weeks.^25^ It is crucial to address bone health by incorporating calcium and Vitamin D3 supplementation while using steroids. Additionally, initiating steroid-sparing therapy with immunomodulators or biologics is part of the plan to minimize prolonged steroid exposure.^6^

The optimal maintenance treatment for patients responding to steroids is not well established. A study by Festa et al. looking at long-term outcomes of ASUC patients found no difference in colectomy rates between those started on aminosalicylates vs. immunomodulators or anti-TNF agents.^26^ Thus, it may be reasonable to commence aminosalicylates for treatment-naïve patients. The key considerations are preventing prolonged steroid exposure through steroid-sparing strategies while also minimizing the risk of relapse through appropriate maintenance treatment.

### Steroid-refractory patients

In cases where patients do not respond to initial IV steroid treatment within 3 days, prompt consideration should be given to second-line agent rescue therapy or surgery. Assessment tools outlined in Table 1 can be utilized to evaluate the response to steroids.

Patients who have not responded to outpatient oral corticosteroids and with a high inflammatory burden at admission may have lower chance response to IVS treatment, suggesting the need for earlier salvage therapy. In an Australian study of 92 admission with ASUC, an albumin on admission < 30 g/L and either corticosteroid therapy over 1 week prior to admission or an endoscopic Mayo score of 3 on flexible sigmoidoscopy identified 32% of the cohort as likely to fail intravenous corticosteroid and require rescue therapy with a PPV of 85%.^27^

Another and important factor to consider in modern UC management is the increased and earlier use of advanced therapies, make a compelling argument for consideration of earlier salvage for such patients admitted with ASUC, who may have already had prior exposure to agents used as salvage therapy.

Ultimately, we need predictors of response to IVS right from admission that are based on parameters that are available or easily accessible for bedside use. In this regard, the ASUC and ADMIT-ASUC scores based on clinical and sigmoidoscopic activity are welcome steps forward and may need to be incorporated into treatment paradigms.^28^^,^^29^

### Second-line agents/medical rescue therapy

For medical rescue therapy, current guidelines suggest the use of cyclosporine or IFX as second-line agents for patients refractory to steroids.^6^^,^^7^^,^^13^

#### Ciclosporin

Ciclosporin, a calcineurin inhibitor, demonstrated its efficacy as a medical rescue therapy for ASUC in 1994. Studies since then, including those by Assche et al., confirmed the effectiveness of both 2 and 4 mg/kg doses.^6^ The short-term and long-term effectiveness and safety of ciclosporin have been extensively explored, with initial clinical response rates ranging from 60 to 80% in various studies.^30^ A recent meta-analysis by Barberio et al. revealed a RR of failure to respond to ciclosporin at 0.45 (CI 0.28–0.76), emphasizing its superiority over placebo.^31^

Reviewing multiple studies, Gisbert et al. noted short- and long-term response rates of ~70 and 52%, respectively, in preventing colectomy with ciclosporin. Another meta-analysis indicated a RR for colectomy with ciclosporin at > 1 month of 0.42 (CI 0.21–0.77), demonstrating its superiority over placebo.^30^ However, no significant difference in response rates compared with placebo was observed over a year (RR 0.72, CI 0.43–1.21).^31^

Upon achieving an initial response, it is advisable to transition to oral ciclosporin as a bridging therapy while progressing towards a more definitive immunomodulator regimen or elective colectomy. Nonetheless, the use of ciclosporin is generally not recommended for patients with an inadequate response to thiopurines, as this is associated with poorer short- and long-term outcomes.^30^ Further studies reveal favourable short-term results when ciclosporin is used as a bridging agent for biologics with slower action and enhanced safety profiles, such as vedolizumab or ustekinumab.^30^ This approach is particularly advantageous for patients with previous exposure to thiopurines and a history of anti-TNF failure.

Tacrolimus, an oral calcineurin inhibitor, has been investigated for cases of steroid-refractory UC, and a recent Cochrane review suggests its superiority (low certainty) over placebo in this context.^30^

#### Infliximab

IFX is a chimeric IgG1 monoclonal antibody that targets free, and membrane bound TNF-α. Jarnerot and colleagues demonstrated evidence of efficacy in a 2005 study, wherein 45 patients with ASUC (4 days after initiating corticosteroids) were administered a single infusion of IFX (5 mg/kg) or placebo. The colectomy rate < 3 months was 29% in the IFX group (7 of 24 patients) vs.67% (14 of 21 patients, *P* = 0.017) in the placebo group.^32^ At 3 years, this effect was maintained with 50% in the IFX group undergoing colectomy vs. 76% in the placebo group, with no IFX given as maintenance.^33^

Optimal dosing of IFX in ASUC remains contentious. The standard induction regime and maintenance doses at 5 mg/kg are licensed for outpatient moderate to severe UC. In ASUC, evidence suggests increased drug clearance and faecal losses resulting in low serum IFX levels, and heightened immunogenicity, leading to therapeutic failure.^34^ Higher CRP, low serum albumin and higher endoscopic severity at admission are linked to failure to respond to IFX.^34^

These factors could have influenced members of the International Organization for the Study of IBD to prefer the use of accelerated schedules or higher doses for induction in ASUC patients, albeit lacking robust evidence.^35^

Chao et al. compared standard induction (5 mg/kg) in 37 patients with ASUC to a 10 mg/kg dose in 35 patients and found no difference in 3-month colectomy rates (5.4 vs. 14.3% respectively, *P* = 0.2).^36^ Govani and colleagues treated 66 patients with ASUC, within IFX (5 or 10 mg/kg) using algorithm driven (CRP to albumin ratio) dosing, with subsequent dosing interval based on CRP levels at Days 3 and 6. There were no significant differences in colectomy rates at 3 months (30.3% with accelerated optimized induction and 24.2% in single dose rescue therapy group, *P* = 0.58).^37^

A meta-analysis by Choy et al. identified that IFX 5 mg/kg multiple-dose induction was superior to single-dose induction but failed to demonstrate the benefit from a dose intensification strategy. However, this should be interpreted in the background that dose intensification was given only to those patients with a higher risk of therapeutic failure.^34^ The PREDICT-UC trial (ClinicalTrials.gov: NCT02770040), a multicentre prospective RCT comparing clinical outcomes with intensified IFX dosing strategy vs. standard induction, should be informative.

From a practical perspective, adverse effects from IFX should be considered before treatment initiation. These include reactivation of latent infections such as tuberculosis and hepatitis and worsen sepsis. IFX can also induce or aggravate heart failure and should be avoided in patients with significant congestive heart failure.^6^

#### IFX or ciclosporin for ASUC

The CYSIF^38^ and CONSTRUCT^39^ trials demonstrated comparable clinical effectiveness for ciclosporin and IFX. Moreover, a recent meta-analysis did not find any difference in colectomy rates or clinical response between the two drugs,^31^ although the long-term cost-effectiveness modelling from CONSTRUCT favoured ciclosporin,^40^ which is less relevant since biosimilar IFX access has reduced cost considerably.

A major consideration for ciclosporin use is its side effect profile(hypertension, nephrotoxicity, electrolyte disturbances, neurotoxicity and infection) and the need for close monitoring of renal function and blood pressure.^30^ Recent network meta-analyses did not note any significant difference in serious adverse events between patients receiving ciclosporin and IFX (RR 0.85, 95% CI 0.49–1.48)^31^ or increase in postoperative complications between both agents.^41^

Nevertheless, the shorter half-life of 7 hr for ciclosporin, compared with 9 days for IFX, could prove advantageous in situations requiring urgent colectomy.

Overall, current evidence suggests that IFX and ciclosporin are equally effective in treating ASUC, with similar rates of clinical remission, colectomy prevention and serious adverse events. The choice between the two medications hinges on individual patient factors, cost considerations and physician preference.

### Sequential therapy

Sequential therapy in the context of ASUC implies the introduction of either ciclosporin or IFX, after the failure of one of these as first-line salvage treatment. In a retrospective study of 9 patients who received cyclosporine after IFX, and 10 patients who received IFX, after cyclosporine, 4 patients achieved remission in the cyclosporin to IFX group(40%), and 3 in the other group(33%, *P* = 0.45).^42^ Serious side effects were reported in 16% patients including one death.^42^ Another retrospective study of 86 patients receiving sequential therapy (cyclosporine followed by IFX), 49 (57%), failed to respond to IFX and went colectomy. Infectious complications were reported in 10% of patients.^43^

A recent comprehensive review incorporating 23 studies assessing sequential therapy found that 53% of patients successfully avoided colectomy despite the observed heterogeneity among the included studies. Notably, treatment sequences involving IFX followed by calcineurin inhibitors, or vice versa, demonstrated comparable treatment outcomes in terms of both efficacy and safety, with colectomy-free rates of 58 and 42%, respectively.^30^

While the evidence on sequential therapy remains contentious, it is essential to weigh the associated risks of serious infection and delays in colectomy, which can contribute to adverse postoperative outcomes. A review noted 26% adverse events with close to a 1% mortality risk, aligning with findings from a previous meta-analysis.^30^ Advocates of sequential therapy argue that this data should be weighed against the ~5% postoperative mortality associated with emergency colectomy.^44^

Due to the insufficient availability of robust evidence and the associated risks of infection and delays in colectomy, guidelines do not currently endorse sequential therapy.^6^^,^^7^ However, it may be considered for well-selected patients in expert.^7^^,^^30^

### Other medical salvage therapies

#### Janus Kinase (JAK) inhibitors

Tofacitinib, a non-selective JAK inhibitor, was the first in class small molecule agent to be approved for induction and remission treatment of moderate to severe UC. Its favourable pharmacokinetics (rapid absorption, reduced vulnerability to drug loss from gut and hypoalbuminemia, and prompt plasma clearance) position it as a theoretically ideal option for patients with steroid-refractory ASUC. Additionally, these characteristics contribute to the potential minimization of complications associated with colectomy, both intraoperatively and postoperatively. Despite its rapid action, the time to respond in patients can range from days to months.^30^

A review of 14 studies, primarily retrospective, assessing response to Tofacitinib in ASUC after failure of ≥1 biologic or ciclosporin revealed a 77% response rate in avoiding colectomy.^30^ A recent systematic review of 21 studies reported a 30-day colectomy-free survival of 85%, a 90-day colectomy-free survival of 86% and a 180-day colectomy-free survival of 69%.^45^

Serious adverse events reported from a review was 4.4%. Tofacitinib's potential concerns, particularly regarding increased cardiovascular and thrombosis risk, were not substantiated in a meta-analysis.^46^

Due to insufficient high-quality studies, tofacitinib is not recommended in guidelines as a rescue therapy for steroid-refractory ASUC and is presently only used off-label. Ongoing trials, TOCASU (ClinicalTrials NCT05112263) and TRIUMPH (ClinicalTrials NCT04925973) in patients with steroid-refractory ASUC who have not responded to IVS are expected to provide more insights into the role of tofacitinib in this context. Simultaneously, a newer selective JAK inhibitor, upadacitinib, has shown promise in ASUC.^47^

#### Vedolizumab and ustekinumab

Due to a relatively slower onset of action, neither of these agents has been exclusively tested in the context of ASUC. They have, however, been used alongside calcineurin inhibitors (ciclosporin and tacrolimus) as a maintenance therapy as an alternative to thiopurines, which can take longer to take effect. Summarizing various studies, the response rate (colectomy avoidance) was 69 and 100% in vedolizumab and ustekinumab, respectively. The included studies were predominantly retrospective and with smaller sample sizes. These drugs may have a place, especially in patients who have previously failed anti-TNF therapy.^30^

### Surgical management

Despite advancements in medical management, proctocolectomy stands asserts its position as ‘curative’ treatment for UC.^48^ Current indications for surgery in ASUC include medically refractory ASUC or cases where medical salvage therapy is contraindicated, massive haemorrhage, toxic megacolon and colonic perforation. Given the increased risk of postoperative complications associated with delayed surgery, it is crucial to involve the surgical team early during ASUC admission, with the goal of performing colectomy within 7 days of refractoriness to medical therapy.^48^ A recent European multicentre cohort study noted an early colectomy rate of close to 10%, which was lower than previous studies, including UK IBD audit data reporting a 17% early colectomy rate in 2010.^26^

When planning surgery for medically refractory ASUC, the procedure of choice is subtotal colectomy and end ileostomy with a long rectal stump, especially in critically ill ASUC patients. Ileal pouch-anal anastomosis (IPAA), if intestinal continuity is desired, is usually performed in two or three stages by an experienced colorectal pouch surgeon. The second and third stages include total colectomy with IPAA and the optional closure of ileostomy during the second stage. These stages are typically performed 3–6 months after the initial procedure and patient recovery. Patients also have the option of undergoing non-restorative proctocolectomy with end ileostomy.^6^^,^^48^

Despite the potential for curative surgery, postoperative pouchitis and wound infections are observed in about half of the patients after restorative proctocolectomy with IPAA. While overall mortality is low at 0.1%, long-term complications such as ileus, faecal incontinence, fistula formation or chronic pouchitis can occur in up to 25%.^48^ Conversely, individuals undergoing non-restorative proctocolectomy face the risk of parastomal hernia and ileostomy prolapse.

Laparoscopic surgery can reduce intra- and postoperative complications, facilitate quicker recovery, result in fewer adhesions and incisional hernias, lead to shorter hospital stays, enhance female fertility and improve cosmetic outcomes, and should be considered when feasible.^48^

## Conclusion

ASUC management is a complex clinical challenge that requires prompt and intensive medical intervention, often involving the expertise of a multidisciplinary team, involving the patient, gastroenterologist, colorectal surgeon, IBD nurse, specialist and stoma therapist. The cornerstone of initial treatment remains corticosteroids, with intravenous methylprednisolone or hydrocortisone being the first-line therapy. For patients who do not respond to corticosteroids, rescue therapies such as cyclosporin or IFX are effective alternatives. Prompt escalation of treatment, including surgery, should be considered early for those who fail to respond to second-line agents to avoid complications. Ongoing research and clinical trials continue to explore the safety and efficacy of newer biologic agents, small molecules and surgical techniques that could positively impact on outcomes for patients with ASUC.

## Data Availability

There are no new data associated with this article. No new data were generated or analysed in support of this research.
